# Association between cannabis use and physical health problems in Norwegian adolescents: a cross-sectional study from the youth survey Ungdata

**DOI:** 10.1186/s12889-022-13136-6

**Published:** 2022-04-06

**Authors:** Ragnhild Mæland, Lars Lien, Marja Leonhardt

**Affiliations:** 1 Faculty of Medicine and Health Science, Norwegian University of Science and Technology, Trondheim, Norway; 2 Norwegian National Advisory Unit on Concurrent Substance Abuse and Mental Health Disorders, Innlandet Hospital Trust, Post Box 104, 2381 Brumunddal, Norway; 3 Department of Health and Social Science, Inland Norway University of Applied Science, Elverum, Norway; 4 Faculty of Health Studies, VID Specialized University, Oslo, Norway

**Keywords:** Cannabis, Adolescence, Physical health, Survey, Norway

## Abstract

**Background:**

Cannabis use is increasing among young Norwegians and several studies show a high incidence of common physical health problems. An association has previously been found between cannabis use and mental health problems. Since physical and mental health problems often co-occur, the aim of this study is to explore the relationship between cannabis use and physical health problems.

**Methods:**

In 2017–2019, the Norwegian youth survey Ungdata collected data from 249,100 Norwegian adolescents, which equalled around 80% of all lower secondary school pupils (13–15 years) and about 50% of upper secondary pupils (16–19 years) in Norway. Descriptive analysis was used to calculate the prevalence of cannabis use and bi- and multivariate logistic regression analysis to examine the association between cannabis use and physical health problems, controlled for sociodemographics and mental health problems.

**Results:**

Almost 10% of Norwegian adolescents had used cannabis once or more in the previous 12 months. The use of cannabis increased with age and it was more prevalent among boys. There is a clear connection between physical health problems and cannabis use (OR = 1.582 (CI: 1.527–1.638)) even after adjusting for sociodemographic variables and mental health problems (OR = 1.366 (CI: 1.312–1.423)).

**Conclusion:**

More studies are needed to explore if there might a bidirectional relationship between cannabis use and physical health problems where physical problems increase cannabis use and cannabis use increases the risk of physical health problems. More knowledge on the effect of and motivation for cannabis use are important for policy makers and health care professionals involved in young people.

## Introduction

Young Norwegians generally feel that they are in good health [1, 2]. However, many are struggling with various health problems, and girls are consistently more affected than boys. For several years, an increase has been observed in the incidence of self-reported mental and physical health problems, which may be due to the steady earlier onset of puberty, the general performance pressure and loneliness among adolescents [3]. The latest population-based youth health survey from Trøndelag, Central Norway (Ung-HUNT4), including adolescents between 13 and 19 years, revealed that around 10% of respondents reported almost daily pain during the previous 3 months. Half of girls and a third of boys had non-specific pain in at least one part of the body, and musculoskeletal pain was most often reported [4]. A fifth reported taking painkillers on a weekly basis, and girls were again overrepresented [1]. In the same survey, 44.5% of girls reported symptoms of anxiety and depression, while the corresponding percentage for boys was 16.5. The proportion with symptoms of anxiety and depression was highest in older adolescents (16–19 years); here more than half of females reported these symptoms [1]. Several studies of adolescents have shown a positive association between mental health problems and alcohol consumption [5, 6]. However, despite an increase in mental illness in young Norwegians, their alcohol use is declining [1].

With regard to cannabis, however, an increase in use has been observed among young people in Norway. In Oslo, the proportion who had used cannabis increased by almost 50% from 2015 (7%) to 2018 (13%) [7]. The harm potential of cannabis is debated, but studies have shown that smoking cannabis is associated with an increased risk of developing various pulmonary diseases such as chronic bronchitis [8] and asthma [9]. Some studies have found a link between cannabis use and cardiovascular disease [10], but other studies are less conclusive [11].

Cannabinoids are sedative in small doses, but in large doses weakly hallucinogenic, which may lead to altered sensory impressions associated with a feeling of confusion and reduced cognitive and psychomotor performance [12]. Cannabis may induce psychosis which is reversed when the cannabis use is ceased. When it comes to the development of schizophrenia there may be a causative link, but this is yet not established. We also know that patients with schizophrenia use cannabis as self-medication to relief depressive states, anxiety and social inhibition. It is the tetrahydrocannabinol part of cannabis that probably induce psychosis [13, 14]. Most studies have explored the relationship between cannabis and depression and a review article from 2014 concluded that cannabis use is associated with a greater risk of developing depression [15]. This is supported by a Norwegian study from 2020 showing that cannabis use was related to later use of antipsychotics, mood stabilizers and antidepressants [16]. A study by Kvitland et al. has shown that the use of cannabis is associated with earlier disease onset and poorer functioning in patients with bipolar disorder [17].

However, an understudied possible risk factor for cannabis use in adolescents is common physical health problems, such as physical pain and nausea. Since there is an association between cannabis use and mental health problems, and between physical and mental health problems [18], it is also important to study the connection between physical health problems and cannabis use. Thus, the aim of the present study is to investigate the prevalence of cannabis use among Norwegian adolescents and to analyse the relationship between physical health problems and cannabis use adjusted for sociodemographic factors and mental health problems.

## Method

### Design

The data analysed in this study come from Ungdata, a cross-sectional survey conducted by the social research institute NOVA at Oslo Metropolitan University [19]. Ungdata consists of a questionnaire for school pupils throughout Norway that contains a compulsory section with 159 questions for lower secondary school pupils (13–15 years) and 168 questions for those in upper secondary school (16–19 years). The questionnaire was completed at the vast majority of secondary schools in the country [19]. Ungdata is offered to all local and county councils in Norway, who administer the questionnaire in collaboration with NOVA and KoRus (regional drug and alcohol competence centres). All data are stored in a national database.

### Sample

Ungdata from 2017 to 2019 contains self-reported data from 249,100 adolescents (149,400 from lower secondary and 99,700 from upper secondary). This means that 80% of all 13–15 year olds in Norway participated in Ungdata during that period. In the age group 16–18 years, about half completed the questionnaire. Respondents were roughly equally divided between boys and girls. The 3 years of lower secondary school were represented by around a third each, but in upper secondary school the first year was overrepresented, having about half of respondents. This is because it is more difficult to administer the questionnaire to older pupils due to exams, work practice and higher drop-out rates [19]. All questionnaires that lacked any of the variables to be used were deleted, giving a final total of 236,963 respondents for the analysis.

### Variables

#### The dependent variable

Cannabis use was investigated with the question “How many times have you used hash/marijuana/cannabis in the past year (12 months)?” Possible responses were “not at all”, “once”, “2–5 times”, “6–10 times” and “11 or more times”. The variable was dichotomized to “not used cannabis in the past year” and “used cannabis once or more in the past year” according to other studies, analysing Ungdata [7, 20]. Those in the latter category are hereafter referred to as cannabis users.

#### Independent variables

For sociodemographic factors, the three variables gender, age and family finances were used. Pupils’ year of secondary school was used to represent age. Socioeconomic status was measured in terms of the variable self-perceived family finances. Participants were asked: “Has your family been well off or badly off in the last two years?”. This variable had the following five response options: “well off all the time”, “mostly well off”, “neither well off nor badly off”, “mostly badly off” and “badly off all the time”.

In Ungdata, the Depressive Mood Inventory was used as a measure of mental health problems, with internal consistency with Cronbach’s α of 0.88. This measure was derived from the Hopkins Symptom Checklist and consisted of the following six parts [21, 22] of the question “Have you been affected by any of the following during the past week?” 1. Felt that everything is a struggle, 2. Had sleep problems, 3. Felt unhappy, sad or depressed, 4. Felt hopeless about the future, 5. Felt stiff or tense, and 6. Worried too much about things. Each part had four response options: “not been affected”, “been affected a little”, “been affected quite a lot”, and “been affected a lot”. The scores for mental health problems were added up and divided by the number of items. The scores ranged from 1 to 4, where 1 equals “not affected” and 4 equals “affected a lot”. It has been estimated that respondents with an average score of 3 or above have a high degree of mental health problems, while those whose average is below 3 have a low degree of mental health problems [23].

Physical health problems were measured by six variables as responses to the question “Have you had any of these problems in the past month?”: 1. Headache, 2. Neck and shoulder pain, 3. Joint and muscle pain, 4. Abdominal pain, 5. Nausea, and 6. Palpitations. Here there were also four possible responses: “not at all”, “sometimes”, “many times”, and “every day”. This variable was dichotomized to “no daily physical health problems” and “daily physical health problems”. Adolescents in the latter category were those who answered “every day” to at least one of the six items [24]. This approach is recommended by the Ungdata project leader to capture severe physical health complaints [25] The six variables as an indicator for physical health problems are used in several waves of the survey, but are not formally validated or reliability tested [26].

### Statistical analysis

Summary statistics were calculated for all variables and differences in physical and mental health problems were analyzed using the Chi-square test. Bi- and multivariate logistic regression analysis was performed to examine the relationship between cannabis use and the independent variables. We controlled the logistic regression analysis for possible confounders such as sociodemographic factors and mental health problems, as these variables are related to physical health [27]. Initially, a robustness test was conducted for cannabis use, dichotomizing the variable into “not at all/once” versus “more than one time”, which resulted in the same results of the logistic regression analysis. Categorical variables were coded as dummy variables for use in logistic regression analysis. Statistical significance was set at *p* < 0.05. Data analysis was conducted using IBM SPSS Statistics for Windows (IBM Corp, Version 27.0, released 2020, Armonk, NY).

### Ethics

Data were obtained from an already established data material (Ungdata survey) collected by the Norwegian Social Research institute (NOVA). The Ungdata survey was administered anonymously online during school hours with a teacher present. The pupils were informed that participation was voluntary, and the data collection was based on informed consent. Norwegian Centre for Research Data (NSD) and NOVA have assessed that the data collection and parents were informed prior to the study (a passive consent scheme). This in line with the privacy protections and regulations. Permission to access and use the data were given by NOVA at Oslo Metropolitan University on the 24.11.2020. All methods were performed in accordance with applicable laws, regulations and research ethics guidelines [28].

## Results

### Characteristics of the study population

Table 1 describes the study population, consisting of 48.8% boys and 51.2% girls. There is a fairly even distribution of respondents between the years of lower secondary and year 1 of upper secondary, but the numbers decline in year 2 of upper secondary and again in year 3. Around 5.2% reported that their family was badly off most or all of the time. A minority reported daily physical health problems, with neck and shoulder pain having the highest percentage at 7.4%. The percentage without any headaches in the previous month was 20.6%, while 7.0% had had a headache every day. When asked if they had felt unhappy, sad or depressed, 40.2% replied no, while 10.7% replied “a lot”.

**Table 1:** Description of the study population (*n* = 236,963)

	***n***	%
**Gender**		
Male	115,581	48.8
Female	121,382	51.2
**Year of secondary school**		
1st year lower sec.	44,740	18.9
2nd year lower sec.	44,239	18.7
3rd year lower sec.	44,208	18.8
1st year upper sec.	44,198	18.7
2nd year upper sec.	35,887	15.1
3rd year upper sec.	23,701	10.0
**Family finances past 2 years**		
Well off all the time	108,135	45.6
Mostly well off	76,442	32.3
Neither well off nor badly off	39,961	16.9
Mostly badly off	9772	4.1
Badly off all the time	2653	1.1
**Cannabis use past 12 months**		
Not at all	217,463	91.8
Once	6867	2.9
2–5 times	6272	2.6
6–10 times	1937	0.8
11 or more times	4424	1.9
**Headache past month**		
Not at all	48,907	20.6
Sometimes	115,114	48.6
Many times	56,404	23.8
Every day	16,538	7.0
**Neck and shoulder pain past month**		
Not at all	85,921	36.3
Sometimes	92,965	39.2
Many times	40,641	17.2
Every day	17,436	7.4
**Joint and muscle pain past month**		
Not at all	115,635	48.8
Sometimes	81,844	34.5
Many times	28,760	12.1
Every day	10,724	4.5
**Abdominal pain past month**		
Not at all	84,815	35.8
Sometimes	101,094	42.7
Many times	41,950	17.7
Every day	9104	3.8
**Nausea past month**		
Not at all	92,282	38.9
Sometimes	106,119	44.8
Many times	31,638	13.4
Every day	6924	2.9
**Palpitations past month**		
Not at all	154,543	65.2
Sometimes	55,062	23.2
Many times	20,256	8.5
Every day	7102	3.0
**Felt that everything is a struggle past week**		
Not been affected	63,658	26.9
Been affected a little	82,034	34.6
Been affected quite a lot	55,099	23.3
Been affected a lot	36,172	15.3
**Had sleep problems past week**		
Not been affected	80,968	34.2
Been affected a little	84,843	35.8
Been affected quite a lot	44,259	18.7
Been affected a lot	26,893	11.3
**Felt unhappy, sad or depressed past week**		
Not been affected	95,376	40.2
Been affected a little	76,162	32.1
Been affected quite a lot	39,976	16.9
Been affected a lot	25,449	10.7
**Felt hopeless about the future past week**		
Not been affected	107,940	45.6
Been affected a little	64,914	27.4
Been affected quite a lot	37,956	16.0
Been affected a lot	26,153	11.0
**Felt stiff or tense past week**		
Not been affected	98,613	41.6
Been affected a little	75,335	31.8
Been affected quite a lot	41,621	17.6
Been affected a lot	21,394	9.0
**Worried too much about things past week**		
Not been affected	62,294	26.3
Been affected a little	67,702	28.6
Been affected quite a lot	60,729	25.6
Been affected a lot	46,238	19.5

### Prevalence of cannabis use

Table 2 shows the prevalence of cannabis users by sociodemographic variables. A total of 19,500 respondents, corresponding to 8.2% of the study population, had used cannabis once or more in the previous 12 months. The figures show that more boys (10.4%) than girls (6.2%) reported having used cannabis. Usage increased with age, from 1.5% in the first year of lower secondary to 18.9% in the final year of upper secondary. Cannabis use shows a steady increase from adolescents whose families were well off all the time (7.3%) to those whose families were badly off all the time (24.0%).

**Table 2:** Prevalence of cannabis use past 12 months by sociodemographic variables (*n* = 236,963)

	***n***	%
**Gender**		
Male	12,018	10.4*
Female	7482	6.2
**Year of secondary school**		
1st year lower sec.	680	1.5
2nd year lower sec.	1408	3.2
3rd year lower sec.	2608	5.9
1st year upper sec.	5067	11.5
2nd year upper sec.	5247	14.6
3rd year upper sec.	4490	18.9
**Family finances past 2 years**		
Well off all the time	7877	7.3
Mostly well off	5755	7.5
Neither well off nor badly off	3832	9.6
Mostly badly off	1398	14.3
Badly off all the time	638	24.0

**Fig. 1:**
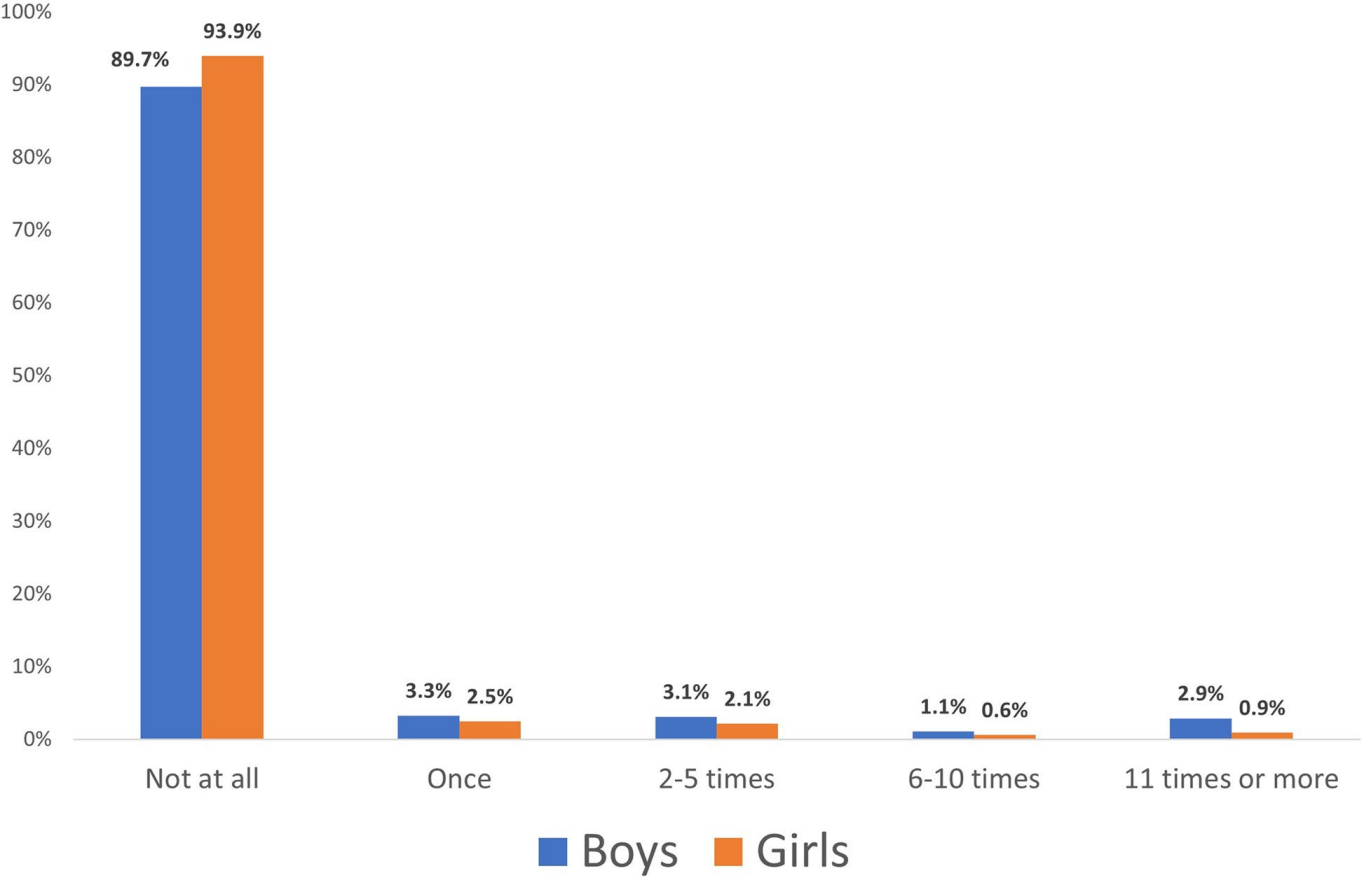
Cannabis use in the past year by gender

We see a gender difference in cannabis use, as illustrated in Fig. 1. Among boys, 89.7% reported not having used cannabis in the previous year, while for girls the figure is 93.9%. For those with high consumption (11 or more times) the figures are 2.9% of boys and 0.9% of girls.

### Physical health problems and cannabis use

Table 3 shows differences in physical and mental health problems between adolescents who had not used cannabis and those who had used cannabis once or more during the previous year. The results show that the cannabis users consistently reported more physical and mental health problems; 10.1% had experienced daily headaches in the previous month, compared to 6.7% of non-users. When comparing cannabis users and non-users, we see greater differences in mental than in physical health problems. A high degree of mental health problems was reported by 16.5% of non-users but 31.6% of users.

**Table 3:** Differences in physical and mental health problems between respondents who have not used cannabis and those who have used cannabis during the past 12 months (*n* = 236,963)

	No cannabis n total = 217,463		Used cannabis n total = 19,500	
	*n*	%	*n*	%
**Headache past month**				
Not at all/sometimes/many times	202,899	93.3	17,526	89.9*
Every day	14,564	6.7	1974	10.1
**Neck and shoulder pain past month**				
Not at all/sometimes/many times	202,259	93.0	17,268	88.6
Every day	51,204	7.0	2232	11.4
**Joint and muscle pain past month**				
Not at all/sometimes/many times	208,172	95.7	18,067	92.7
Every day	9291	4.3	1433	7.3
**Abdominal pain past month**				
Not at all/sometimes/many times	209,613	96.4	18,246	93.6
Every day	7850	3.6	1254	6.4
**Nausea past month**				
Not at all/sometimes/many times	211,709	97.4	18,330	94.0
Every day	5754	2.6	1170	6.0
**Palpitations past month**				
Not at all/sometimes/many times	211,549	97.3	18,267	93.7
Every day	5869	2.7	1233	6.3
**Mental health problems**				
Low degree	181,504	83.5	13,332	68.4
High degree	35,959	16.5	6166	31.6

^*^All statistically significant relationships, *p* < 0.05

### Association between health problems, sociodemographic factors and cannabis use

The results of the logistic regression analysis are presented in Table 4. The unadjusted correlations in Model 1 show a significant association between cannabis use and physical health problems (OR = 1.582; CI 1.527–1.638), and mental health problems (OR = 2.360; CI 2.286–2.437). When adjusting for sociodemographic variables (Model 2), the odds ratio increased to 1.744 (1.678–1.813), and when controlling for mental health problems (Model 3), the odds ratio declined to 1.366 (1.312–1.423).

**Table 4:** Association between different explanatory variables and reported cannabis use past 12 months (*n* = 236,963)

	Model 1 Unadjusted OR (95% CI)	Model 2 Adjusted OR (95% CI)	Modell 3 Adjusted OR (95% CI)
**Physical health problems** (reference: no daily problems)			
Daily problems	1.582 (1.527–1.638)	1.744 (1.678–1.813)	1.366 (1.312–1.423)
**Gender** (reference: male)			
Female	0.567 (0.550–0.584)	0.467 (0.452–0.482)	0.408 (0.395–0.422)
**Year of sec. School** (reference: 3rd year upper sec.)			
1st year lower sec.	0.066 (0.061–0.072)	0.065 (0.060–0.070)	0.070 (0.065–0.076)
2nd year lower sec.	0.141 (0.132–0.150)	0.134 (0.126–0.143)	0.141 (0.132–0.150)
3rd year lower sec.	0.268 (0.255–0.282)	0.255 (0.243–0.269)	0.260 (0.247–0.274)
1st year lower sec.	0.554 (0.530–0.579)	0.520 (0.497–0.543)	0.531 (0.508–0.556)
2nd year lower sec.	0.733 (0.702–0.766)	0.694 (0.664–0.725)	0.706 (0.675–0.739)
**Family finances past 2 months** (reference: badly off all the time)			
Well off all the time	0.243 (0.222–0.266)	0.325 (0.295–0.359)	0.411 (0.372–0.454)
Mostly well off	0.251 (0.229–0.275)	0.336 (0.305–0.371)	0.411 (0.372–0.454)
Neither well off nor badly off	0.326 (0.297–0.358)	0.410 (0.371–0.453)	0.479 (0.433–0.530)
Mostly badly off	0.516 (0.465–0.573)	0.581 (0.520–0.650)	0.632 (0.565–0.708)
**Mental health problems** (reference: low degree of mental health problems)			
High degree of mental health problems	2.360 (2.286–2.437)		2.219 (2.137–2.305)

*OR* odds ratio, *CI* confidence interval

Model 1 shows unadjusted bivariate correlations between cannabis use in the previous 12 months as the dependent variable, and physical health problems as an independent variable

Model 2 examines the association between cannabis use and health problems adjusted for gender, age and family finances (SES), while Model 3 is also adjusted for mental health problems

## Discussion

Almost 10% of secondary school pupils had used cannabis once or more in the previous year. Usage increased with age and was most common among boys. Further, there is a statistically significant association between physical health problems and cannabis use among Norwegian adolescents, even after adjusting for gender, age, self-reported family finances and mental health.

With its large sample, Ungdata presents a good cross-section of Norwegian youth and the vast majority had not used cannabis in the previous 12 months. When comparing this with figures from the European School Survey Project on Alcohol and Other Drugs (ESPAD), we see that Norwegian adolescents use less cannabis than those in several other countries. The Czech Republic had the highest prevalence of adolescent cannabis use in Europe at 28%, with Italy close behind with 27% [29]. The difference in cannabis use may be related to the fact that fewer young people in Norway smoke tobacco than in most other European countries [29]. Tobacco and cannabis use are closely linked [30], and thus a non-smoker of tobacco is less likely to start smoking cannabis. Another possible explanation is that Norway has long had a restrictive cannabis policy where its use is criminalized. Only a few European countries mention cannabis use in legislation, and around one-third of EU countries have in practice decriminalized the possession of small quantities of cannabis for personal use [31].

About 7% reported having neck/shoulder pain and headaches daily. This is a somewhat lower figure than in the Ung-HUNT study, where 10.2% reported musculoskeletal pain almost daily. The difference may be because Ung-HUNT used different response categories; their figures would probably have been lower if they had only included respondents reporting daily pain [4].

The present study shows that cannabis use increases with age, which is in line with international research [32], and that cannabis use decreases with increasing socioeconomic status. Studies from Norway show that the youngest users came from families with low socioeconomic status, while the opposite is true for older users [7]. Research from other countries shows somewhat varying results with regard to cannabis use and socioeconomic status. A French study found that young people with high socioeconomic status have tried cannabis more often than those with low socioeconomic status [33] and Hasin et al. found that lower socioeconomic status leads to more cannabis use [34], which supports the findings in the present study.

### Health problems and cannabis use

The results from this study show that adolescents who had used cannabis in the previous 12 months had more physical health problems than those who had not used cannabis, in terms of both nausea and pain. There may be a number of reasons for this. Cannabis has been shown to cause nausea, especially in people who have used it for a long time and in large doses [35]. However, research also shows good evidence that cannabis has a positive effect on nausea, and a great deal of research has been conducted on cannabis as a treatment for chemotherapy-induced nausea [36]. This may be transferable to nausea that occurs for other reasons, but here there is little research. It is therefore possible that the association found in our study is because adolescents experiencing nausea take cannabis to alleviate the problem, but nausea arising from cannabis use cannot be discounted either.

The results also show that the cannabis users reported more pain in the form of headaches, abdominal pain and musculoskeletal pain. It is said that there is moderately good evidence that cannabis has a pain-relieving effect [37, 38]. It is thus possible that cannabis is used as self-medication; pain would then lead to cannabis use rather than the opposite relationship. There is still too little knowledge about the types of pain where cannabis has the most positive effect, but neuropathic pain in adults has been well studied [39]. It is thus less clear whether cannabis relieves headaches, abdominal pain and musculoskeletal disorders in adolescents.

We found a clear association between cannabis use and physical health problems in the bivariate analysis, and in regression analysis when controlling for gender, year of secondary school and socioeconomic status in addition to mental health problems. This suggests a real connection between cannabis use and physical health problems. What is more, young adults (18–25 years) may use cannabis as self-medication to reduce physical and mental distress [40], and further, it has been shown, that pain relief is the primary motivation for cannabis users with chronic pain [41]. The causal relationship has, however, not sufficiently been studied among adolescents as cannabis use may increase the risk of physical health problems, and physical health problems may be a risk factor for cannabis use.

There exist different models for the relationship between substance use and mental health problems; these may be transferable to the relationship between substance use and physical health problems. The first theory is the self-medication hypothesis. This implies that people with physical health problems use cannabis to treat them, and the use is then secondary to the health problems. This is a popular theory, but has little support in research [42]. Another possible explanation for the association between physical health problems and cannabis use is the harm model [43]. This implies that cannabis use leads to or triggers health problems that might not otherwise have developed, and that the symptoms will diminish if cannabis use ceases. A third explanatory model is the common factor model, according to which there are one or more factors that increase the risk of developing both physical health problems and cannabis use. These can be psychological, social or genetic factors.

### Strengths and limitations of the study

Ungdata has a number of strengths; it is population-based with a large sample and a high response rate, thus reducing the likelihood of selection bias.

The questionnaire consists of a large number of questions, which enables the examination of a variety of relationships while also controlling for possible confounders. Pupils from secondary schools throughout Norway participated, which makes the results representative of the Norwegian secondary school population. The study also has its limitations. Ungdata is a cross-sectional study and no conclusions can be drawn about causal relationships. The cannabis variable was dichotomized to either “have not used cannabis in the past 12 months” or “have used cannabis once or more in the past 12 months”. The latter response thus covers a wide range from those who had only tried it once to those with high regular consumption. The use of cannabis shows a strongly skewed distribution; it is commonly assumed that a relatively small group accounts for more than two-thirds of use [44]. The variable is broad and categorizes an inappropriate number of adolescents as “cannabis users”. This can make our results inaccurate and difficult to interpret. What is more, in Norway cannabis use is criminalized. Thus, assessing cannabis use within a school-survey may lead to an underreporting of the actual cannabis use prevalence, due to the fear of being identified and so punished for cannabis use [45]. Although the Ungdata survey is anonymous, this underreporting might be common especially in regions with small adolescent populations.

It is important to be aware that all the Ungdata results are based on self-reported data. It is not entirely clear whether the adolescents understand the questions in the same way as the researchers and whether they answer truthfully. This would imply low validity. Many of the questions are about cannabis use, behaviour and health problems over the past 12 months. It may well be difficult for some respondents to remember details about what took place so far back in time, which can lead to information bias if, for example, cannabis users do not remember as well as non-users.

### Practical implications

The association between cannabis use and physical health problems in adolescents can be useful knowledge for anyone who works with this age group. It could be especially useful for physicians and nurses in secondary schools and health centres for adolescents to enable them to detect physical health problems in cannabis users. If health professionals working with adolescents become more aware of the connection between physical health problems and cannabis use, they may find it more natural to ask adolescents whether they use cannabis when they come to be treated for physical health problems. It will also place healthcare workers in a better position to inform young people about the acute and chronic harmful effects of cannabis use to enable them to make informed choices about whether they want to take the risks involved. Our study reveals both, that cannabis use may lead to physical health problems, or that physical health problems may lead to cannabis use. Therefor it is important that health professionals examine the context around cannabis use among their adolescent patients to provide an integral treatment and follow up.

## Conclusion

The aim of this study was to investigate the association between cannabis use and physical health problems in Norwegian adolescents. The results indicate a real connection between cannabis use and physical health problems, but the causal relationship needs further study. This makes it important for healthcare workers to pay particular attention to the physical health of cannabis users and to inform adolescents to a greater extent about the possible harmful effects of cannabis. Further research is clearly needed in this field, and qualitative analyses can provide greater insight and understanding of the causal relationships between cannabis use and physical health problems.

## Data Availability

The Ungdata set is available for researchers on request to NSD - Norwegian Centre for Research Data.
